# A New Evaluation Tool to Obtain Practice-Based Evidence of Worksite Health Promotion Programs

**Published:** 2008-09-15

**Authors:** Diane O Dunet, Phillip B. Sparling, James Hersey, Pamela Williams-Piehota, Mary D Hill, Michele Reyes, Carl Hanssen, Frances Lawrenz

**Affiliations:** Division of Nutrition, Physical Activity, and Obesity, National Center for Chronic Disease Prevention and Health Promotion, Centers for Disease Control and Prevention; Georgia Institute of Technology, Atlanta, Georgia; RTI International, Research Triangle Park, North Carolina; RTI International, Research Triangle Park, North Carolina; Centers for Disease Control and Prevention, Atlanta, Georgia; Centers for Disease Control and Prevention, Atlanta, Georgia; Hanssen Consulting, Ada, Michigan; University of Minnesota, Minneapolis, Minnesota

## Abstract

**Introduction:**

The Centers for Disease Control and Prevention developed the Swift Worksite Assessment and Translation (SWAT) evaluation method to identify promising practices in worksite health promotion programs. The new method complements research studies and evaluation studies of evidence-based practices that promote healthy weight in working adults.

**Methods:**

We used nationally recognized program evaluation standards of utility, feasibility, accuracy, and propriety as the foundation for our 5-step method: 1) site identification and selection, 2) site visit, 3) post-visit evaluation of promising practices, 4) evaluation capacity building, and 5) translation and dissemination. An independent, outside evaluation team conducted process and summative evaluations of SWAT to determine its efficacy in providing accurate, useful information and its compliance with evaluation standards.

**Results:**

The SWAT evaluation approach is feasible in small and medium-sized workplace settings. The independent evaluation team judged SWAT favorably as an evaluation method, noting among its strengths its systematic and detailed procedures and service orientation. Experts in worksite health promotion evaluation concluded that the data obtained by using this evaluation method were sufficient to allow them to make judgments about promising practices.

**Conclusion:**

SWAT is a useful, business-friendly approach to systematic, yet rapid, evaluation that comports with program evaluation standards. The method provides a new tool to obtain practice-based evidence of worksite health promotion programs that help prevent obesity and, more broadly, may advance public health goals for chronic disease prevention and health promotion.

## Introduction

The workplace setting is an important venue for health promotion practices among adults because most working-age men and women are in the workplace most days (1). Worker safety and health insurance benefits are focal points both for the Centers for Disease Control and Prevention (CDC) and for employers. CDC's National Institute for Occupational Safety and Health is the key federal entity working to prevent employee injury and illness, and CDC partners with the business community as part of an overall strategy to achieve public health goals for adult health and quality of life through prevention (2). For example, as employers increasingly sought to promote employee health and reduce their own burden of health care costs through worksite health promotion programs, CDC partnered with the National Business Group on Health to develop *A Purchaser's Guide to Clinical Preventive Services* (3).

Obesity affects employee health care costs (4), productivity (5), absenteeism (6,7), injury (8), and chronic diseases (9). Well-executed studies reported in the peer-reviewed scientific literature provide an evidence base for designing worksite health promotion programs that may decrease the prevalence of obesity and related chronic diseases. CDC undertakes rigorous, systematic scientific reviews of published studies on obesity prevention and control in conjunction with the Task Force on Community Preventive Services, which publishes the *Guide to Community Preventive Services* (10). CDC has also systematically reviewed secondary literature sources, such as dissertations and evaluation reports, to identify potentially promising practices that merit further attention and formal study.

Worksites that provide health promotion programs to their employees are another source of information about interventions that may be effective in addressing obesity. To better understand such programs, CDC developed the Swift Worksite Assessment and Translation (SWAT) method to assess worksites rapidly and to add to the body of what has been called "practice-based evidence" (11). Research studies require strict adherence to protocols that often intentionally hold an intervention static over time to control threats to study validity. However, organizations may create new health promotion strategies or adapt and customize research-based strategies to the demographics and health status of their workforce, available resources, or other factors. Current events and news media that focus on such issues as obesity also can stimulate new ideas.

Through professional networking and sources such as worksite health promotion award programs, CDC staff regularly become aware of "home-grown" interventions that are reputed to achieve desired health outcomes. An evaluation method is needed, however, to investigate these types of emerging practices, particularly to identify practices that merit more comprehensive, rigorous (and expensive) evaluation research. Worksite health promotion strategies already being implemented by employers are obviously feasible, in contrast to research-based interventions that may not necessarily be adaptable to myriad workplace settings.

Most research studies of corporate worksite health promotion programs involve large companies. However, more than 70% of adults in the US workforce are employed in organizations with fewer than 5,000 employees (12). Smaller organizations may have fewer resources available for evaluation; however, they may have the ability to change their policies or programs rapidly, unencumbered by the administrative systems of large organizations. The SWAT method was designed specifically for evaluating organizations we defined as small (<300 employees) and medium-sized (<5,000 employees) because of the paucity of data in these settings. The method has since been adapted for large worksites.

Within this worksite health promotion context, we developed the SWAT approach as a middle-ground evaluation method that aims to be business-friendly while being solidly based in good evaluation practice. SWAT is not meant to quantify the success of worksite health promotion practices but rather to assess quickly whether they are worthy of more rigorous evaluation. This article discusses the process used to develop and test SWAT, describes the SWAT method in detail, discusses the program evaluation standards used to refine it, and briefly summarizes the conclusions of an outside evaluation of the SWAT method.

## Methods

Beginning in June 2005, the project team of researchers from CDC and RTI International identified nationally recognized experts in worksite health promotion, site-visit methods, and evaluation. Fourteen experts from academic, corporate, worksite health promotion, and public health settings were invited to consult on the project (see Acknowledgments).

During the next 3 months, the project team developed an initial SWAT framework (Table 1). The work was guided by CDC's Framework for Program Evaluation in Public Health (13), the centerpiece of which is nationally recognized program evaluation standards (14). The overall SWAT framework explicates inclusion and exclusion criteria for worksite programs, operational definitions, site selection criteria, and overall procedures for conducting site visits. The 14-member expert panel was invited to provide additional feedback and recommendations on the SWAT framework in writing or by telephone conference at 2 critical points during the framework development process.

The SWAT development project plan was reviewed by the associate director for science of CDC's National Center for Chronic Disease Prevention and Health Promotion, who determined it to be public health practice and not research subject to institutional review board approval for human subjects.

A notable feature of the SWAT development process is that the SWAT evaluation method was concurrently evaluated by Western Michigan University's Evaluation Center (WMUEC) (15). The SWAT meta-evaluation is summarized in Table 2. The SWAT framework was further refined based on the process recommendations and summative findings of the WMUEC evaluation team.

The SWAT evaluation method was designed as a tool for single-worksite assessments and uses a set of criteria (Table 3), rather than a comparison group, to judge the promise of worksite health promotion practices. CDC funding was available to conduct 9 initial SWAT site visits. Because these assessments were the first application of the SWAT method, they informed further refinement of the SWAT process. A detailed description of the initial 9 worksites assessed for their innovative practices is reported elsewhere in this issue of the journal (16).

As shown in Table 1, the SWAT evaluation process is organized into 5 phases: 1) site identification and selection, 2) site visit, 3) evaluation of health promotion practices, 4) evaluation capacity building, and 5) translation and dissemination. The overall approach of a SWAT assessment is a document review process and an on-site examination of self-reported summary data from worksites, supplemented by key informant interviews and observation. The combination of evaluation methods provides a general understanding of the worksite health program and its major components, measured outcomes, and data quality.

After potential SWAT sites were identified by using methods described in Table 1, the site selection process began with a brief, structured telephone interview to compile a preliminary summary of a site. This information was used to determine site eligibility and the potential for a SWAT assessment to identify a promising practice. Table 4 presents the eligibility criteria we used to limit the types of worksites considered for our initial group of assessments. The initial 9 sites ranged in size from 115 to 3,200 employees and included manufacturing, construction, health care, higher education, and government organizations.

The criteria presented in Table 3 were applied to determine whether a site appeared to be implementing a promising practice related to obesity or healthy body weight. As described below, these same criteria were the basis of the post-visit assessment to evaluate whether a practice or program was judged to be promising.

An alternative to the SWAT evaluation approach would be for a site visit to be conducted by a "connoisseur" — that is, an expert with deep subject matter knowledge, extensive site-visit experience, perceptual sensitivities, and refined insights. Limitations to this approach are 1) its inherent subjectivity and 2) the dearth of connoisseurs who can evaluate emerging, innovative strategies (17). Although a site visit is at the heart of the process, SWAT differs from a connoisseur evaluation because it uses 3 graduated levels of worksite health promotion and evaluation expertise (Table 1). In a SWAT evaluation, connoisseurs (Level 3) make a summative evaluation judgment about whether a practice is promising, an assessment for which such high-level expertise is crucial.

To promote consistency in site-visit observations and to reduce subjectivity, SWAT protocols include interview guides and written templates. For example, site visitors used a data review template to guide their written inventory of the types of data that sites collected and the kinds of routine analyses performed (Table 1, Phase 2, Step 8). Another example is a structured checklist used by the site visitor to conduct an observational environmental assessment of the worksite facilities and the surrounding community, including such factors as distances to local restaurants and availability of places to engage in physical activity.

In exchange for hosting a CDC site visit, worksite staff members were provided opportunities to receive CDC technical assistance throughout the SWAT process. The first opportunity arose naturally during the site visit, for example, as employers interacted with site visitors to complete an inventory of their data collection and routine program evaluation activities. At one site, the site visitor noted that the employer's existing data could be used to assess employee participation by job category or health risk factor. Analysis of these data could be used to plan recruitment strategies to engage hard-to-reach employees, especially those who might stand to derive the greatest health improvements.

A comprehensive site-visit report (approximately 15 pages), which was developed by the site-visit team, followed a structured template that explicitly addressed each of the SWAT criteria described in Table 3. A draft site-visit report was shared with each site to verify the report's accuracy and to give sites the opportunity to add relevant information. Each site also received written technical assistance in the form of a 3- to 4-page "interpretive assessment" developed by members of the project team who are CDC staff and Level 3 connoisseurs. The interpretive assessment highlighted the site's practices that were particularly notable and suggested improvements in the site's health promotion program and evaluation methods. In formulating these site-specific suggestions, the CDC experts were guided by a detailed outline of recommended practices for worksite health promotion programs  (Table 5). To avoid overwhelming a site with a long list of recommendations, the authors of the interpretive assessments considered the site's available resources and readiness to change, and limited suggestions to those that seemed readily achievable. Each site also was offered a 1-hour telephone consultation with CDC project team experts to discuss the report, the interpretive assessment, and any other topics related to worksite health promotion (Table 1, Phase 4, Step 11). The WMUEC team also conducted brief post-visit telephone interviews with worksite staff to ascertain what benefits the sites perceived from participation in the SWAT project and to take suggestions to further improve the SWAT process.

A tenet of the SWAT approach is that the business and public health communities can learn from each other. During the 12 months after the initial 9 participating sites were evaluated, CDC hosted a series of 1-hour telephone conferences approximately every 2 months as a way to foster peer-to-peer networking and to further build these sites' capacities, especially with regard to using evaluation methods to strengthen programs. The technical assistance topics presented included how to increase program participation, strengthen the collection and use of data, and develop a business case for worksite health promotion.

The SWAT method is built on CDC's Evaluation Framework (13), which has as its core the program evaluation standards of utility, propriety, accuracy, and feasibility (14), developed by the Joint Committee on Standards for Educational Evaluation. With the help of the meta-evaluation feedback, the SWAT project team considered the standards throughout the development process and documented decisions in meeting minutes and file notes. We report the outcomes of the SWAT development process and its success in meeting our goal for SWAT to be business-friendly, effective for our public health purpose in identifying promising practices, and appropriate in its balance of the program evaluation standards of utility, propriety, accuracy, and feasibility.

## Results

To ensure that the SWAT method was business-friendly as well as practical (the utility standard), it was designed to be shorter and less costly than a rigorous evaluation study while still generating sufficient data to assess promising practices. A cornerstone of the SWAT approach is to establish a collegial relationship with each organization and to minimize the burden on employers so that site visits will be welcome. This approach was not only beneficial in the site-visit stage but also has led to continuing collegial relationships. For example, 8 sites volunteered to participate in CDC worksite health promotion activities, including interviews for a CDC educational video.

As a scientific agency, CDC wanted accurate data (the accuracy standard). Accuracy was balanced against the feasibility of accessing data under nonresearch conditions (the feasibility standard). Few, if any, business organizations engage in health-related data collection that meets the rigorous standards of a scientific study. Instead, organizations routinely collect and analyze data for business processes to understand and track employee benefits, health insurance costs, absenteeism, and safety. Recognizing that tradeoffs would be necessary, we used 2 approaches to balance the feasibility of data collection with the adequacy and accuracy of data.

First, site visitors used a template to guide their review of each site's in-house data, including data quality and data analysis activities. Second, site visitors elicited examples of strategies that worksite staff thought were particularly successful based on their in-house data. For example, one site shared summary data that tracked participation before and after an incentive policy change from a cash bonus to paid time off, showing a dramatic increase in employee enrollment in the health promotion program.

Ensuring data accuracy is particularly challenging in a practice-based approach because key definitions may vary widely among worksite staff, health promotion experts, and public health evaluators. In contrast to a research study in which terms are carefully defined, the initial 9 sites we visited described program success in different ways, such as program participant satisfaction, improvements in employees' health risk factors, and savings realized through reduced health insurance expenditures. To address the evaluation standard of accuracy and to strengthen the rigor of our approach, we developed written definitions of key terms, especially those related to program outcomes.

Federal privacy rules on individual-level health data dictated that we limit our interviews to key informants acting in their official capacity, such as a company president or worksite health promotion coordinator (the propriety standard). Individual employees/program participants were not interviewed. However, to better understand the employee perspective, site visitors conducted a group interview with an employee wellness committee, if one existed, since these employees were acting in their official role as committee members. Site visitors asked employers to share only summary data and were vigilant in ensuring that they did not view individual data even when sites offered to share it.

Another opportunity to hear from site staff after the site visit was the 1-hour telephone conference calls (Table 1, Phase 4, Step 11). Several sites reported that they implemented new strategies or changed their practices on the basis of the suggestions in CDC's interpretive assessment. For example, 3 sites had taken steps to provide improved access to healthier foods in their cafeterias and vending machines, and 4 sites instituted new data analyses to enhance their tracking of employee participation and to customize recruitment efforts to attract nonparticipating employees.

A SWAT assessment is not designed to draw a conclusion about the effectiveness of particular strategies. Such an assessment would not be possible because SWAT site visitors do not collect or analyze individual-level data, nor do they verify accuracy of analyses shared by employers. To identify practices that hold promise for further, more rigorous evaluation, the SWAT approach relies on the judgment of Level 3 connoisseurs. In this case, 3 CDC staff who were not part of the project team independently reviewed the site-visit reports and identified practices that were promising.

The SWAT project intentionally sought innovative programs as a counterpoint to more established programs reported in the peer-reviewed literature. However, a reality of practice-based evaluation is that organizations frequently change strategies to keep them fresh and interesting. To address this variability, site visitors focused on obtaining information on 1) key program practices, especially those that were continued over time, and 2) program practices that were related to weight control or weight loss, which was consistent with the project's goals.

The separate efficacy evaluation of SWAT conducted by WMUEC (Table 2) concluded that SWAT's strengths were its systematic and detailed procedures, its service orientation (ie, its feedback to worksites and its pursuit of public health goals), and the accuracy of the information it provides. Potential weaknesses in the SWAT method were identified by WMUEC throughout the process, allowing the project team to revise and improve the approach. Remaining weaknesses identified (Table 2) further highlight the challenges in conducting practice-based evaluation. The 3 benefits of participation most commonly cited to the WMUEC team at the post-visit evaluation were the opportunities to 1) reflect on their program, 2) learn from other organizations, and 3) be associated with CDC. Suggestions for improving SWAT emphasized the need to convey the purpose of the project at multiple points with multiple site staff.

SWAT is intended to be more rapid and less costly than full-scale, rigorous evaluation. On the basis of project records and application of the SWAT method subsequent to our initial 9 sites, we estimate the number of hours for one SWAT assessment as follows: 87 hours for Level 1 evaluators; 34 hours for Level 2 experts; and 4 hours for Level 3 experts. The time from initial contact with a potential site through the technical assistance conference call requires a minimum of 2 months and an average of 4 months.

## Discussion

Well-designed, well-executed research studies may provide the best possible scientific information; however, absent such studies, the best available information can still be useful in making progress toward public health goals (18). Furthermore, evaluation of evidence-based practices can be supplemented by accumulation of practice-based evidence from the evaluation of programs and strategies being implemented in the field. CDC identified a need for an evaluation method that can be deployed more rapidly than a research study but that provides a more rigorous assessment than programs' self-reports.

The limitations of the SWAT method in large part correspond to the SWAT criteria for assessing promising practices. We sought innovative programs; however, innovators were likely to innovate continually, making "program" difficult to define. Data quality was another SWAT criterion used to assess programs; however, CDC and RTI site visitors neither collected nor analyzed primary data related to the worksite health promotion program participants or their health status and outcomes. Instead, we relied on summary data provided by worksites to generally assess the site's data quality and use of data for its program evaluation.

Using practice-based evidence for evaluation presented challenges not only to CDC but also to worksites. For example, employers faced obstacles in obtaining employee health data for longitudinal analysis of health outcomes without compromising the confidentiality of employees' private information. Some sites hired vendors to handle employee data, and others limited their data collection to repeated cross-sectional measures of indicators such as the blood pressure status of their entire workforce or body weight measurements of weight loss team members.

A contribution of a practice-based approach to evaluation is the potential to shape a research agenda by identifying the practices that appear to be most promising and thus worthy of future investments in rigorous research that can measure their effect precisely. As researchers design a new intervention to study, they can look to practice-based evaluations to better understand how employers implement their programs and strategies in a wide range of worksite settings and for diverse workforces. Such evaluations can yield feasible strategies that can be broadly disseminated. Thus, evidence-based and practice-based approaches can work together to broaden the knowledge base as we address public health problems.

Although the SWAT approach was designed for small and medium-sized worksites to assess health promotion programs that may affect adult obesity, CDC has already adapted this method for large worksites and expanded it to include other chronic diseases and preventive health areas. An extension of the SWAT approach recently used by CDC was for a large employer to fund a SWAT assessment of 3 of its business units' worksite health promotion programs, policies, benefits, and environmental supports. As part of this partnership, CDC conducted an assessment of the worksite program, with the understanding that the employer will implement several evidence-based strategies that CDC recommends based on the high-priority health issues identified through the assessment. Thus, CDC has new resources and an avenue to expand its practice-based evidence on worksite health promotion conducted in a natural setting.

The SWAT method has also been used to inform CDC's knowledge base related to community environmental and policy strategies for childhood obesity prevention. A new project, funded by a large foundation and in partnership with CDC, is sponsoring 60 SWAT-type assessments through 2008. Finally, CDC is using the SWAT method as the basis for developing a comprehensive self-assessment tool for employers. Similar to the School Health Index (http://apps.nccd.cdc.gov/SHI/Default.aspx), a new Workplace Health Index will guide employers in reviewing their programs, practices, and policies that promote employee health.

The SWAT development process generated a middle-ground evaluation method that is business-friendly and effective in guiding rapid assessment of potentially promising practices in worksite health promotion. An independent evaluation of the SWAT method concluded that the method is acceptably compliant with the program evaluation standards of utility, propriety, accuracy, and feasibility and is effective in providing data sufficient for experts in health promotion to identify promising and innovative worksite health promotion strategies. A strength of SWAT is that it makes explicit how promising practices are determined, thus reducing the subjectivity of such determinations.

The public health response to the need for worksite health promotion strategies to address obesity includes both practice-based evidence and evidence-based practice as complementary sources of information. As a practice-based evidence approach, SWAT assessments are especially useful in providing preliminary information to guide future investments in research studies that can more rigorously examine those practices that appear most promising. Furthermore, insight into worksite health promotions can help researchers design interventions with the potential for broad dissemination, creating a loop from practice to research and back to practice.

## Figures and Tables

**Table 1:** Steps in the Swift Worksite Assessment and Translation (SWAT) Project Process to Assess Worksite Health Promotion Programs and Required Level of Expertise to Evaluate Steps, 2005

**Phase**	**Required Evaluator Level^a^**
**Phase 1. Site Identification and Selection**	
1. Potential worksites are identified through Internet searches, nominations from health promotion experts, award programs, and word of mouth.	Levels 1, 2, and 3
2. Existing documents from sources such as corporate Web sites are reviewed to assess eligibility for SWAT assessment.	Levels 1 and 2
3. Invitation letter sent from CDC to worksite inviting participation in a SWAT assessment and requesting additional information about the health promotion program.	Level 1
4. For those worksites that accept the invitation, documents provided by the worksite are reviewed and summarized using a template. Special note is made of strategies specifically intended to address obesity as well as the in-house data collection and evaluation that the site undertakes.	Level 1
5. To fill in gaps in the summary template (step 4, above), a brief telephone interview is conducted with health promotion staff.	Level 1
6. SWAT project team reviews program information in summary template.	Level 2
7. Using the criteria shown in Table 3, the team decides whether to conduct a site visit.	Levels 2 and 3
**Phase 2. Site Visit**	
8. One-day site visit divided as 2 half-day observations is conducted by a team of 2 to 3 evaluators. Written protocols guide interviews with senior managers and health promotion program leaders and staff. A written observational environmental checklist is completed to note worksite conditions such as fitness facilities, cafeteria and vending, signage, and stairwells. An additional written environmental observational checklist provides context on the surrounding community, including distance to restaurants and availability of places for physical activity.	Level 1
9. Comprehensive site-visit report is written following a prescribed template.	Level 1
**Phase 3. Evaluation of Health Promotion Practices**	
10. SWAT project team members and connoisseurs review site-visit reports to identify innovative and promising practices. Written assessment form is used to guide assessors' consideration of SWAT criteria regarding what constitutes a "promising practice." These experts justify their selection of promising practices.	Levels 2 and 3
**Phase 4. Evaluation Capacity Building**	
11. SWAT project team members develop an interpretive assessment based on health promotion practice criteria. Written interpretive assessment and final site-visit report are provided to sites.	Level 3
12. One-hour telephone consultation provided to sites to discuss interpretive assessment and provide technical assistance as desired.	Levels 2 and 3
**Phase 5. Translation and Dissemination**	
13. Innovative and promising practices are shared with CDC researchers and managers to inform planning for future investments in research studies.	Levels 2 and 3
14. Innovative worksite strategies are disseminated to the business community through ongoing telephone conferences with SWAT alumnae sites, CDC networks, and other CDC-business partnerships.	Level 2
15. Communication materials, including online video of health promotion practices, are used to disseminate information to business audience at large.	Level 2

a Level 1, skills in interviewing and observation using written protocols and general knowledge of evaluation principles and practice. Level 2, formal training and experience in conducting evaluation and designing or implementing worksite health promotion programs. Level 3, leadership of evaluation studies in worksite health promotion and extensive knowledge of evaluation, evidenced by publication of journal articles, book chapters, or texts.

**Table 2:** Summary of Independent Meta-Evaluation of the Swift Worksite Assessment and Translation (SWAT) Project^a^

Category	Criteria
**Key questions**	What are the strengths and weaknesses of the SWAT process, including each of its components in terms of producing its intended results? How can SWAT be improved? How effective is SWAT for producing its intended results? To what degree does the SWAT framework enable an evaluator to produce an evaluation that satisfies accepted program evaluation standards?
**Key activities**	Before any site visits are conducted, convene an independent expert panel to duplicate the SWAT rating process to assess its efficacy. Review SWAT documents, including protocols and rating forms. Provide "just-in-time" feedback to CDC's SWAT methods development team, especially with regard to the program evaluation standards of the Joint Committee on Standards for Educational Evaluation. "Observe" weekly project telephone calls and attend 4 of the initial 9 SWAT site visits to observe implementation of SWAT protocols. Conduct telephone interviews with SWAT site visitors, project team members, and staff from the sites visited. After the initial 9 site visits, the expert panel stages a duplicate review of the SWAT documents (1-page program summary and full site-visit reports) to assess which steps of the SWAT process are essential in order to judge promising practices.
**Key findings**	SWAT is an effective method for rapid evaluation of worksite health promotion programs. Sites reported that SWAT was well-organized but took longer than expected. Site staff reported that the SWAT reports, phone calls with the SWAT project team, and dissemination activities were accurate, useful, and of high quality. The primary benefit that accrued to the sites was that SWAT forced sites to be introspective about their programs. Site personnel reported that the nature of the questions and the site-visit protocols required them to think about both their program and their evaluation practices. For some sites, this took the form of an "internal audit," which might not have occurred without SWAT.
**Strengths of SWAT process**	Used a systematic approach based on recognized evaluation principles and practices. Adhered to program evaluation standards. Had adequate and accurate data, especially considering short amount of time for collection. Included worksite key staff as stakeholders in reviewing and interpreting SWAT site-visit reports and conclusions. Had a service orientation, both to serve public health goals and to provide feedback to worksites on ways to enhance or improve their programs and program evaluation.
**Recommendations to strengthen SWAT process**	CDC staff should personally communicate with worksite staff to ensure that the purpose of the project and the site visits are fully understood. Reduce bias by interviewing both participants and nonparticipants in worksite health promotion as key informants in the SWAT protocols. Streamline interview protocols to eliminate redundancy in questions. Shorten the time from initial contact with a site to a site visit and reporting.

a Conducted by Western Michigan University Evaluation Center, Kalamazoo, Michigan (15).

**Table 3:** Criteria Used to Assess Worksite Health Promotion Practices for the Swift Worksite Assessment and Translation (SWAT) Project

Category	Criteria
**Innovativeness**	Is the practice new or different from evidence-based recommendations? Is it a substantial variation/improvement on an existing effective practice?
**Data quality**	How valid, reliable, and convincing are the data used by the worksite to assess healthy weight outcomes? Priority will be given to higher-quality data, especially measured (vs self-reported) height and weight.
**Effectiveness**	How successful is the practice in helping adults achieve and maintain a healthy body weight? Is there evidence of impact on eating patterns or physical activity? On health outcomes? On absenteeism?
**Sustainability**	*Sustainability of health outcomes.* During what period have employees maintained weight loss or healthy weight (or improved nutrition and physical activity health habits)? *Program sustainability*. How sustainable is the practice over time?
**Public health relevance**	To what degree is the practice consistent with public health ethical and practice standards (eg, noncoercive, safe)? Would the practice be appropriate to post on the CDC Web site?
**Feasibility**	To what extent does the practice seem feasible for replication in other worksites (especially small worksites)? What is the potential for dissemination of this practice to other settings?

**Table 4:** Criteria to Assess Worksite Eligibility for the Swift Worksite Assessment and Translation (SWAT) Project


**Worksites must:**

1. Have data to document the success of the program, where success is defined as one of the following for at least a 1-year period: Weight loss among overweight participants (body mass index [BMI] ≥25.0 kg/m^2^) or favorable changes in eating patterns or physical activity and prevention of weight gain among participants at a healthy weight (BMI ≥18.5 kg/m^2^ and <25.0 kg/m^2^).
2. Be willing to consider CDC suggestions for enhancing the design of their program evaluation activities.
3. Agree to publicly share, through CDC channels, information about their worksite health promotion practices.

**Programs must:**

4. Have valid, reliable, and convincing data used by the worksite to assess healthy weight outcomes. Priority will be given to higher-quality data, especially measured (vs self-reported) height and weight.
5. Be conducted in a workplace or a community surrounding a workplace.
6. Be sponsored by a US company/organization that has been in operation for at least 3 years. (Interventions for military personnel are excluded. Interventions for civilians in a military setting may be included.)
7. Be open to participation by most employees. (Executive-only type programs are excluded.)
8. Operate at the worksite for a minimum of 1 year.
Programs may be at an individual, group, organizational (virtual/physical), or community level and may involve behavior, policy, or environmental changes.

**Table 5:** Criteria Used to Develop Interpretative Assessments for the Swift Worksite Assessment and Translation (SWAT) Project

Category	Criteria
**Program goals and components**	
**Goals**	Program design is clearly articulated. Program activities are logically related to goals. Participants understand the goals of the program. Management and program staff share same goals.
**Components**	Practices offered are tailored to workforce(s). Both nutrition and physical activity practices offered. Program provides feedback on participant progress. Program offers reinforcement to employees — that is, use of incentives, encouragement by staff, and social support. Practices appear acceptable or appealing to employees. Program has effective coordination with medical services.
**Reach and participation**	All employees are eligible for program. Participation rates are similar for men and women, among different cultural groups, by all levels and types of employees. Spouses and family members are eligible and participate. Participation rates are meeting program's goal. Definition of participation is satisfactory.
**Policy supports** (refers to organizational policies that support a "culture of wellness")	No-smoking policies. Healthy food at meetings. Release time to exercise. Health care incentives.
**Environmental supports**	Exercise/fitness areas and locker rooms. Stairwells or other building design features to promote exercise. Outside walking/cycling paths and bike racks. Break rooms/refrigerators/microwaves. Vending machines/cafeteria offering healthy foods. Signage, posted newsletters, and e-mail messages promoting participation.
**Community supports**	Strong health-promoting organization-community partnerships. Community environmental support (eg, bike/walking trails, parks). Community-based medical/health events/initiatives. Public education/social marketing efforts. State health department support/resources.
**Sustainability**	Management support is evident. Program has an active wellness planning/advisory committee. Health promotion integrated throughout corporate culture. Program costs likely to provide return on investment.
**Program tracking and evaluation**	
**Program delivery**	Tracking of interventions. Tracking of employees (ie, participation and intensity). Tracking of program costs.
**Key measurements** (specific measures are keyed to specific program elements, tactics, or strategies)	Health risk appraisal. Biomedical — measured weight, height, waist circumference, blood pressure, cholesterol. Behavioral — diet and physical activity. Subjective — participants' satisfaction and engagement. Fiscal — costs for facilities, staff, and incentives. Other measurements on specific changes or effects of strategies (eg, change in vending machine options, new pedometer program).
**Data collection process**	Data are stored and managed with easy-to-use database technology. Staff are skilled in data management and retrieval. Individual data are collected systematically. Data are collected at meaningful intervals. Entry allows for reporting both cross-sectionally and longitudinally. Entry allows for reporting by category (eg, sex, age, job category). Program staff review reports regularly.
