# Erratum, Vol 5: Issue 4

**Published:** 2008-12-15

Table 1 in the article "A New Evaluation Tool to Obtain Practice-Based Evidence of Worksite Health Promotion Programs," which appeared in the October 2008 issue, was amended by renumbering the steps. Now 15 steps are included in description of the Swift Worksite Assessment and Translation (SWAT) project process. The changes were made October 29, 2008, and appear online at http://www.cdc.gov/pcd/issues/2008/oct/07_0173.htm. We regret any confusion or inconvenience this error may have caused.

